# Ontology-conformal recognition of materials entities using language models

**DOI:** 10.1038/s41598-025-03619-y

**Published:** 2025-05-28

**Authors:** Sai Teja Potu, Rachana Niranjan Murthy, Akhil Thomas, Lokesh Mishra, Natalie Prange, Ali Riza Durmaz

**Affiliations:** 1 Group of Meso and Micromechanics, Fraunhofer Institute for Mechanics of Materials IWM, 79108 Freiburg, Germany; 2 IBM Research Europe Zurich, Rüschlikon, 8803 Switzerland; 3 University of Freiburg, 79098 Freiburg, Germany

**Keywords:** Materials science, Fatigue, Large Language models, Named entity recognition, Parameter-efficient fine-tuning, Foundation models, Prompt engineering, Ontology, Literature mining, Materials science, Computer science, Mechanical engineering

## Abstract

Extracting structured and semantically annotated materials information from unstructured scientific literature is a crucial step toward constructing machine-interpretable knowledge graphs and accelerating data-driven materials research. This is especially important in materials science, which is adversely affected by data scarcity. Data scarcity further motivates employing solutions such as foundation language models for extracting information which can in principle address several subtasks of the information extraction problem in a range of domains without the need of generating costly large-scale annotated datasets for each downstream task. However, foundation language models struggle with tasks like Named Entity Recognition (NER) due to domain-specific terminologies, fine-grained entities, and semantic ambiguity. The issue is even more pronounced when entities must map directly to pre-existing domain ontologies. This work aims to assess whether foundation large language models (LLMs) can successfully perform ontology-conformal NER in the materials mechanics and fatigue domain. Specifically, we present a comparative evaluation of in-context learning (ICL) with foundation models such as GPT-4 against fine-tuned task-specific language models, including MatSciBERT and DeBERTa. The study is performed on two materials fatigue datasets, which contain annotations at a comparatively fine-grained level adhering to the class definitions of a formal ontology to ensure semantic alignment and cross-dataset interoperability. Both datasets cover adjacent domains to assess how well both NER methodologies generalize when presented with typical domain shifts. Task-specific models are shown to significantly outperform general foundation models on an ontology-constrained NER. Our findings reveal a strong dependence on the quality of few-shot demonstrations in ICL to handle domain-shift. The study also highlights the significance of domain-specific pre-training by comparing task-specific models that differ primarily in their pre-training corpus.

## Introduction

Design of materials and components is influenced by numerous factors and process parameters. Distributed across extended process chains, these variables determine the resulting properties and functional performance. Effectively, this culminates in a vast design space requiring effective management. To navigate it effectively, latent knowledge on composition-process-microstructure-property (CPMP) relationships embedded in scientific literature must be better leveraged. A key step is the extraction of this information into structured semantic formats. This study focuses on materials fatigue, a subdomain where structured information extraction is particularly valuable due to the inherently time-consuming and costly nature of its experimental characterization.

One challenge in extracting coherent structured information is that related data is scattered across the text, tables, and figures of multiple scholarly articles referencing each other^1,2^. To address this challenge, methodologies are required to extract and link structured information from various unstructured sources. In principle, knowledge graphs are flexible data representations which can model such relationships between data scattered within and across articles. In materials science, multiple approaches were proposed based on large language models (LLMs) to extract materials data and build knowledge graphs^3–5^. Another requirement for structured information extraction is that retrieved information is consistently annotated with respect to each other and previously generated datasets, making data interoperable. This is challenging since data reported in scholarly articles is not only very heterogeneous but also often incomplete and sometimes ambiguous. This is owed to the fact that authors are primarily aiming to publish small excerpts of the underlying experimental work to underpin specific novel findings rather than comprehensive datasets^6^. However, most aforementioned knowledge graph generation approaches did not sufficiently account for the data heterogeneity and ambiguity that can be present in articles. Instead, the algorithms and models can decide on arbitrary entity types and relationships that are neither explicitly defined nor conformant to a standard, which limits the interoperability of derived knowledge graphs with other datasets.

Considering the ambiguity present in scholarly articles, approaches are necessary for the disambiguation of entities and relations using various contextual and multimodal cues to reliably assign entities to explicitly defined types. Further, to tackle data heterogeneity, the defined types need to be related to each other through domain knowledge expressed in a formal language. These objectives could benefit from ontologies which are formal, shared conceptualizations of domains^7^. Ontologies allow for incorporating domain-specific knowledge as axioms in description logic. This renders such knowledge machine-interpretable, effectively enabling logical reasoning, improving semantic interoperability, and providing utility for data harmonization. Thus, when structured extraction of information is concerned, a common demand is that it should conform to an existing ontology^8^. Adhering to a pre-defined ontology allows for merging and reuse of datasets—which were previously semantically-annotated in rigorous efforts—in new contexts to address a variety of different objectives. However, to comply with the demand of conforming to a pre-defined ontology, a language model needs to reliably assign a class label to entities in text. This so-called named entity recognition (NER) is a fundamental subtask when transforming unstructured information contained in text into structured knowledge and the main scope of the work at hand.

Pre-trained language models based on the transformer architecture^9^ achieve state-of-the-art performance when fine-tuned on token-classification tasks such as NER. Language models are typically pre-trained using self-supervised learning on large unlabelled text corpora. During pre-training, they capture patterns connecting words in the training corpus. When these models are used for a NER downstream task, they are extended with a classification head, and the model is fine-tuned using an annotated NER dataset. In practice, models like BERT^10^, RoBERTa^11^, and DeBERTa^12^ have become popular for named?entity recognition. To better serve specific fields, versions of BERT have been customized with domain-specific training data. For instance, SciBERT^13^ is optimized for the biomedical sector using scientific texts, while MatSciBERT^14^ focuses on materials science articles. Fine-tuning of language models can be computationally expensive depending on the model size. This challenge has driven researchers to explore and develop more efficient fine-tuning methods, known collectively as parameter efficient fine-tuning (PEFT)^15^. A notable PEFT method is low-rank adaptation (LoRA)^16^, which simplifies the model’s weight updates into lower-rank matrices. Techniques like LoRA have proven to be especially effective in scenarios with limited data, making them particularly appealing for fields like materials science, where annotated datasets are often scarce^17^.

Nowadays, LLMs continue to grow in model size and are trained with sophisticated training procedures on ever larger quantities of diverse data. This renders toady’s language models more flexible and imbues them with the capability of adhering to tasks and demonstrations specified in a prompt. Such models are also referred to as foundation language models and have shown advanced capabilities in generative natural language processing across various tasks including machine translation^18^, question answering^19^, reasoning^20,21^, spurring the development of autonomous language model systems such as agentic systems^22^. In theory, a major advantage of foundation LLMs is that they are comparatively domain-agnostic and require less tuning towards the target domain and task. This makes them attractive for the materials science field where there is a plethora of tasks, only few of which are supported with sufficiently large annotated datasets. In contrast to foundation models, information extraction models based on conventional language models are typically highly task-specific^23^. However, it was previously observed that foundational LLMs have difficulties in assigning entity labels in NER reliably, especially in domain-specific settings^24^. This was attributed to the fundamental mismatch between the sequence labeling nature of NER and the primary focus of LLMs on text generation^25^. Generative LLMs excel in predicting the next word in a sequence based on contextual clues, prioritizing fluency, grammatical correctness, and coherence in generated text over capturing subtle semantic clues crucial for accurate entity recognition^26,27^. Efforts to bridge this chasm between NER and LLMs have encouraged innovative approaches. Researchers have explored strategies such as incorporating explicit entity-tagging instructions into prompts^28^ and hybrid architectures combining sequence labeling and text generation^29^.

**Fig. 1:**
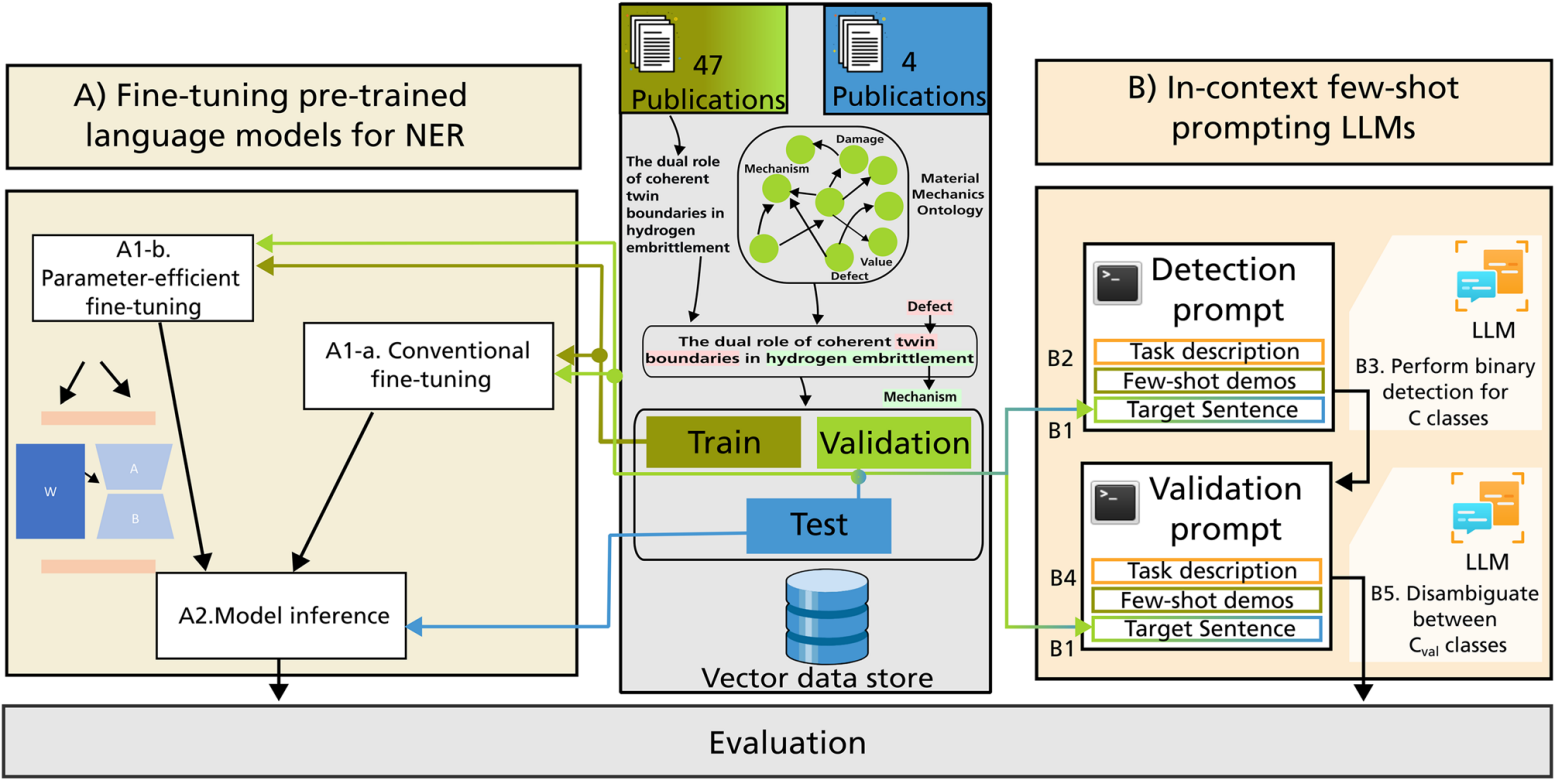
The figure depicts the workflow followed in this work, where the arrows indicate the flow of data. Two sets of publications were segmented into sentences, annotated using class labels from material mechanics ontology, encoded using an embedding model, indexed using a vector store, and partitioned into train, validation, and test sets. These subsets were then used for fine-tuning specialized NER models in branch A and within a few-shot LLM prompting approach in branch B as indicated by the arrows. The former approach A does not utilize the sentence embeddings. In the latter approach, B1 indicates the sequential sampling of target sentences from the test or validation set and B2 the fetching of $k_1$-closest few-shot demonstrations from the training set. Both are used to populate a detection prompt template which is then passed to the LLM to obtain class-annotated entities for the target sentence (B3). Subsequently, for each class within the set of candidate classes $\text {C}_{val}$ predicted for the same entity during detection, a set of $k_2$-closest few-shots are fetched (B4) and then passed to the LLM for validation (B5).

This work assesses whether the domain- and task-agnostic characteristics of foundation LLMs can be exploited to alleviate the data sparsity issue in the materials science domain. In particular, we focus on domain-specific NER to assess how effectively LLMs can identify and classify entity types specific to the materials science field, as defined in the materials domain ontology. Our main approach compares the performance of foundational models using few-shot in-context learning (ICL) against language models that have been fine-tuned for specific tasks. The comparison covers conventional fine-tuning of multiple pre-trained BERT variants, their LoRA counterparts, and two GPT LLM-variants. The utility and potential of these distinct methodologies in materials science are discussed. The considered data covers CPMP relationships and has an emphasis on materials fatigue. The wide range of influence factors in materials fatigue establishes the demand for fine-grained recognition of many specific entity types. This is especially challenging for all-purpose LLMs as it requires distinction of very domain-specific entity types which hold a similar meaning, yet are potentially rather underrepresented in the LLM’s pre-training corpus. To retrieve all relevant influence factors of materials fatigue, considering full-texts rather than only abstracts is imperative. Another objective is to assess the generalization capabilities of task-specific language models and LLM foundation models with respect to typical subdomain shifts. To do so, the model performance is evaluated on two datasets, where one is sampled from the same documents as the training samples/few-shot demonstrations, and the other is sampled from separate publications. The scope of this out-of-distribution dataset is microstructure-property relationships with a weaker emphasis on fatigue.

The primary contributions of this work can be summarized as follows:

- Providing quantitative evidence demonstrating the limitations of foundation models in capturing fine-grained, materials domain-specific semantics, and highlighting the usefulness of domain-aligned pretraining for high-fidelity scientific information extraction.
- Demonstrating the influence of domain shift and the superior generalization capability of fine-tuned task-specific language models compared to in-context learned LLMs by evaluating on samples which are in-distribution (ID) and out-of-distribution (OOD) with respect to their fine-tuning domain or few-shot examples.

In addition, the work also makes the following contributions:

- Development of a two-stage in-context learning pipeline for structured entity extraction, offering a novel and cost-effective alternative to full-model fine-tuning in low-resource, high-specialization domains.
- Analyzing the ICL strategy critically for foundation LLM-based NER in a domain-specific setting and deriving methodological improvement opportunities.
- Comparing conventional fine-tuning models with low-rank adaptation for varying domain gaps between pre-training and downstream domain.

## Results

This work compares task-specific fine-tuning of smaller LLMs (Fig. 1 branch A) with ICL of foundation LLMs (Fig. 1 branch B) on a NER task in the materials fatigue domain. The datasets, algorithms, and tools are described thoroughly in the Methods section and summarized in the following paragraphs. The MaterioMiner dataset^30^ acts as the test dataset on which the NER approaches are compared. An additional dataset was created by annotating text extracted from 47 peer-reviewed publications in the materials fatigue domain. This dataset was further split into a training and a validation set. All datasets are based on the materials mechanics ontology^31^, which amongst others describes the named entity types as classes. While the same annotation guidelines were followed to create the datasets, they exhibit distinct data distributions, specifically different distribution of annotated classes and a small domain shift. Complementary to the stronger emphasis on materials fatigue in the train and validation set, the scientific articles that contribute to the test set have additionally a focus on microstructure-property relationships.

**Fig. 2:**
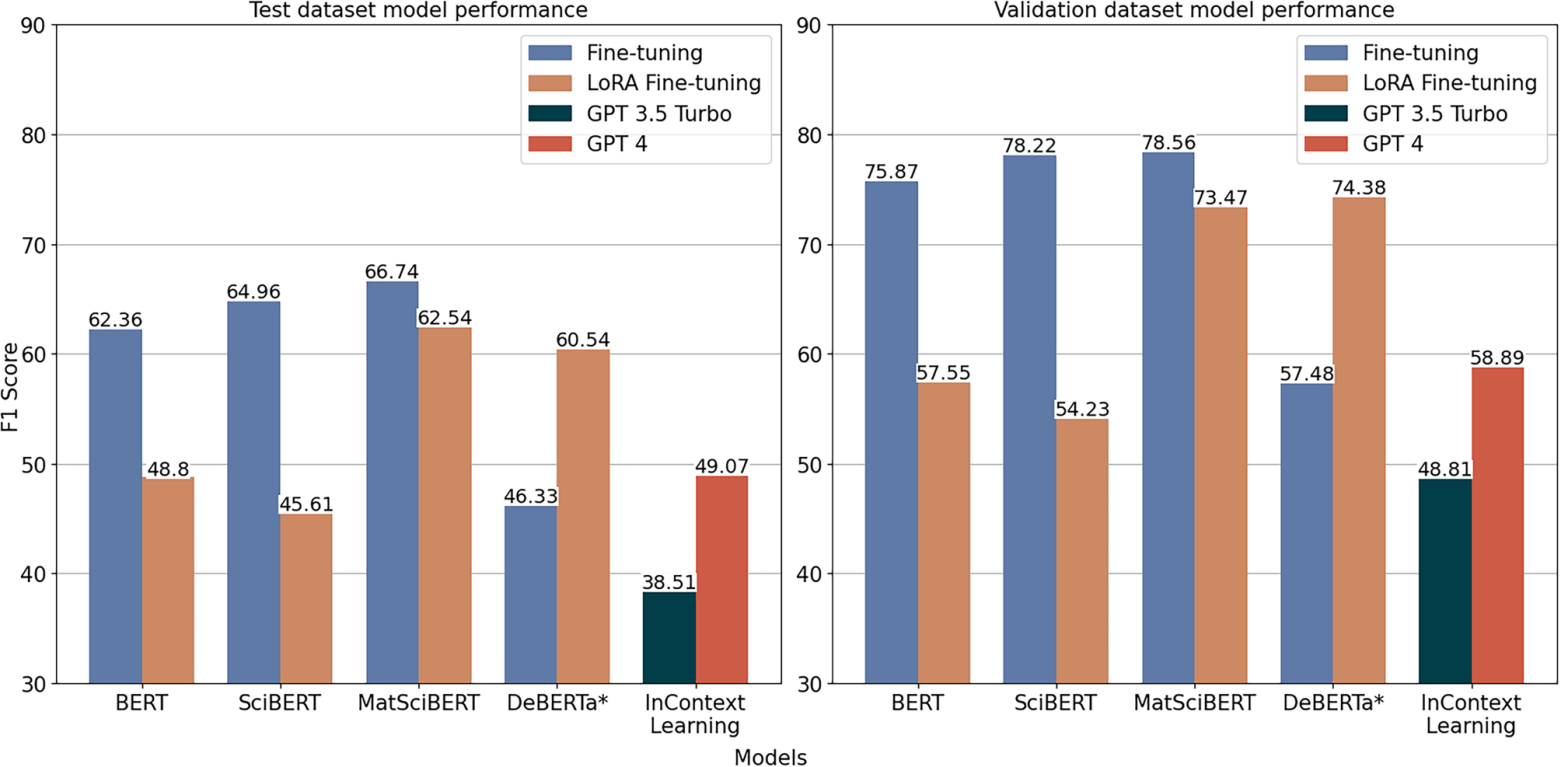
The figure compares the performance of fine-tuned specialized NER models and in-context learning with large language models on a NER task in materials fatigue domain. For each specialized NER model, the mean F1-score achieved on validation and test datasets across five random initializations are presented. The BERT, SciBERT, and MatSciBERT models (distinct model initializations) were fully fine-tuned while only the last layer of the DeBERTa model (distinct architecture) was fine-tuned. Additionally, parameter-efficient fine-tuning was also performed for these models using LoRA. ICL of GPT-4 and GPT-3.5-turbo models were performed.

ICL of GPT-3.5-turbo and GPT-4 were conducted in this study. Few-shot examples were created from the training set and the performance was evaluated on the MaterioMiner dataset. The specialized NER models fine-tuned were BERT-based with a token classification head. Different pre-trained states of BERT^10^ were fine-tuned: BERT-BASE (uncased)^10^, SciBERT Sci-vocab (uncased)^13^, and MatSciBERT^32^. Further, an architectural modification of BERT and a larger model, DeBERTa^12^, was fine-tuned. By default, the entire model was fine-tuned except for DeBERTa, where only the last layer was fine-tuned. Moreover, the conventional fine-tuning of models was compared against a PEFT approach called LoRA^16^. For performance assessment, we use the F1-score. All training and evaluation specifics are detailed in the Methods Section.

From Fig. 2, it can be observed that all three BERT-based task-specific language models when fine-tuned conventionally performed better than ICL of foundation LLMs, while the partly fine-tuned variant of DeBERTa falls short. Furthermore, the LoRA fine-tuned variants of MatSciBERT and DeBERTa also outperformed the foundation LLMs. The best fine-tuned model based on MatSciBERT achieved a relative increase in performance of 36% and 33% on test and validation datasets respectively, when compared to the ICL of GPT-4. However, it is to be noted that fine-tuning used the whole training dataset while only a few systematically selected examples are provided in the LLM prompt for ICL. Thus, it is noteworthy that with the limited few-shot examples, GPT-4 achieved close to 60% F1-score on the validation dataset.

**Fig. 3:**
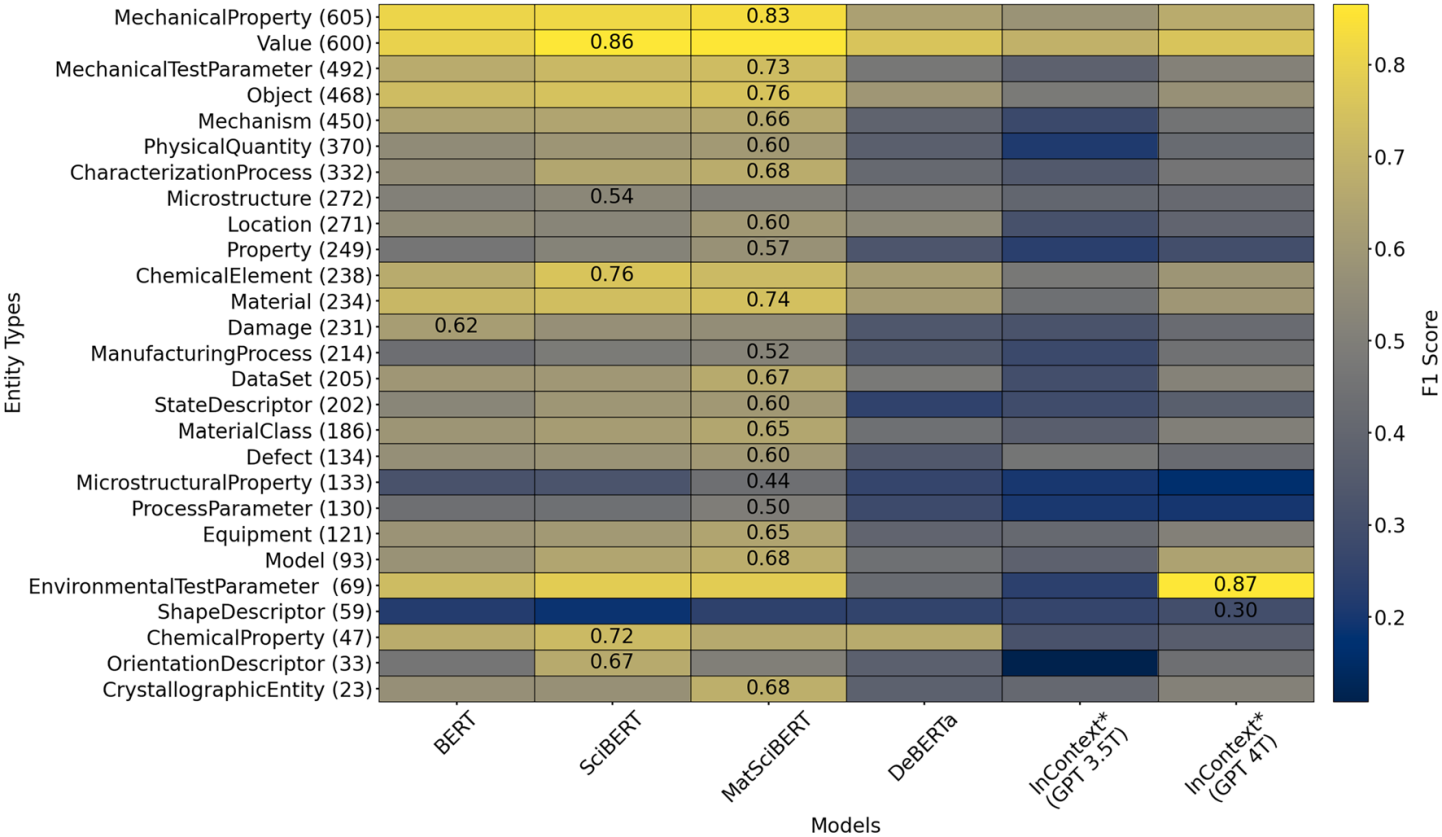
A heatmap of class-wise F1-scores on the test set for the different models are presented. Aside from the color-coding, for each entity type (row), the highest F1-score is stated. The figure shows the test F1-score for each class. The number of training instances for each entity type is denoted inside the parentheses next to it. The asterisk symbol (*) denote that the in-context learning approaches use a fixed number of demonstrations (k = 10) from the training set.

Comparing the performance of different task-specific LLMs presented in Fig. 2, it can be seen that the MatSciBERT-based model consistently outshines others in both test and validation sets. It performs better than the SciBERT-based model, which itself surpasses both the standard BERT and the DeBERTa models. Notably, the DeBERTa model, fine-tuned only on its last layer, has a drop in performance by approximately 20% when compared to the best-performing MatSciBERT model.

Regarding PEFT, we can observe a downgrade in performance from conventional fine-tuning to LoRA fine-tuning of BERT variants. The performance of LoRA BERT and LoRA SciBERT decreased by approximately 15–20% compared to their fully fine-tuned models. The LoRA MatSciBERT variant also showed a reduced F1-score, however, the value was comparatively closer to its conventionally fine-tuned reference. In contrast, LoRA DeBERTa shows a pronounced performance improvement compared to the last-layer fine-tuning of DeBERTa scoring the best F1-score on the validation set among the LoRA models.

**Fig. 4:**
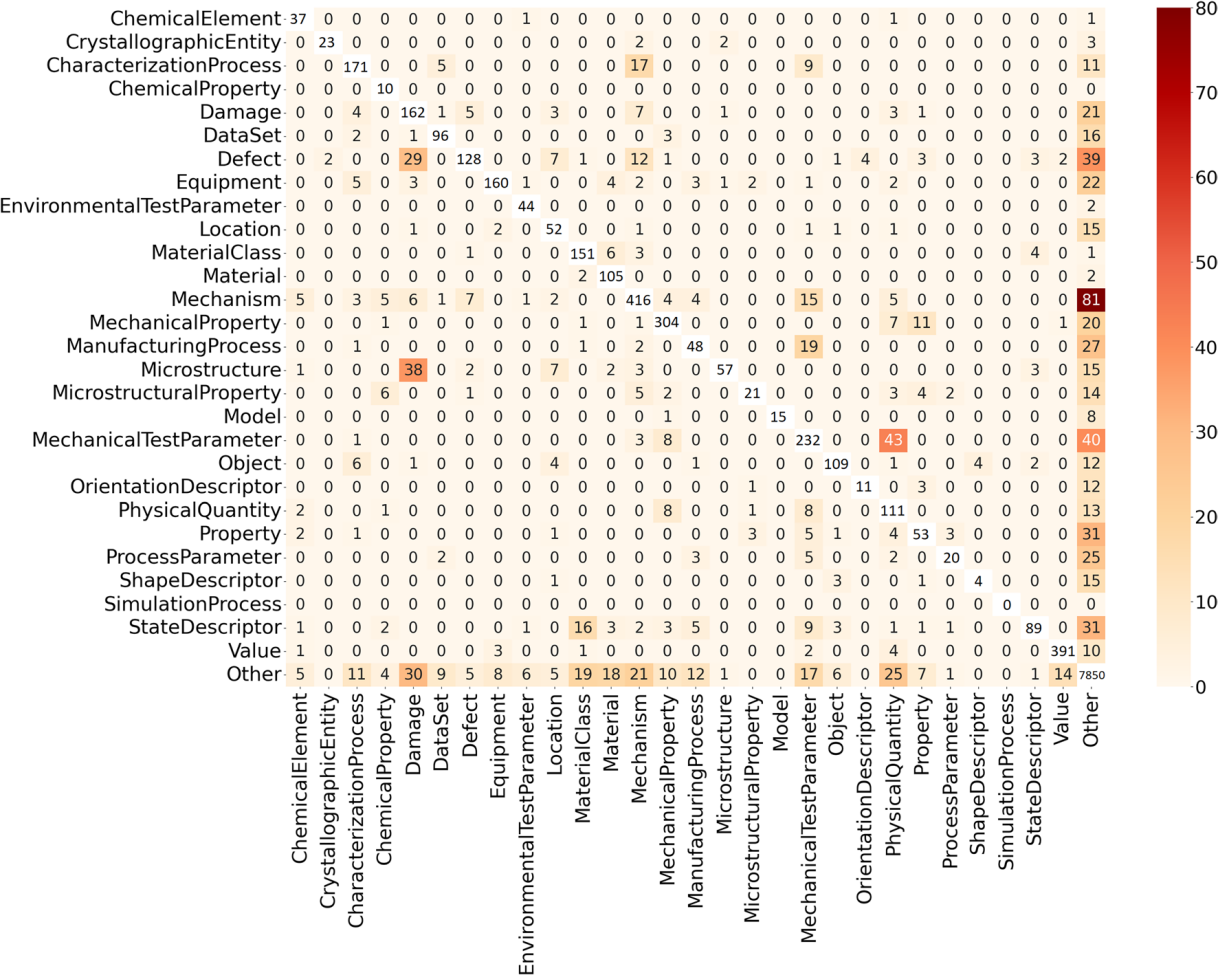
A confusion matrix for the test data where the vertical and horizontal axes are the label and MatSciBERT-based model prediction respectively. The absolute values, indicating the number of confusions between two classes, are only color-coded for the off-diagonal elements to visually highlight relevant confusions.

Additionally, it can be observed from Fig. 2 that the performance of all models on the validation dataset (in-distribution performance) is much better than their performance on the test dataset (out-of-distribution performance). All models show an in-distribution to out-of-distribution performance decrease ranging between 14 and 20%. This indicates that all the models presented do not generalize well to out-of-distribution examples. Among fine-tuning strategies, better generalization performance was observed for LoRA fine-tuning compared to full fine-tuning. For instance, the full fine-tuned BERT exhibited a performance decline of 18% while the corresponding LoRA fine-tuning showed a decrease of only 15%. Additionally, the generalization performance of ICL was observed to be worse than that of task-specific models—with the in-context GPT-4 declining by 17% compared to the conventionally fine-tuned MatSciBERT, showing a decline of 15%.

A heatmap of the class-wise average F1-scores of each model for the test dataset is presented in Fig. 3. The highest F1-score value for each entity type is additionally mentioned in the corresponding column. The rows are sorted by number of instances in the training dataset (numeric values in parentheses contained in the tick labels). With a few exceptions, the fully fine-tuned MatSciBERT-based NER model reaches the best performance for the majority of entity types, mostly in domain-specific entity types such as “Defect”, “Damage”, or “CrystallographicEntity”. SciBERT claims the best F1-scores in most of the remaining types, followed by in-context GPT-4 and BERT. Overall, “Value”, “MechanicalProperty”, and “EnvironmentalTestParameter” types yielded the best results. Further, all models performed poorly on “ShapeDescriptor” and “MicrostructuralProperty”. For entity types which are supported with few training instances, task-specialized models fall sometimes short of ICL.

Figure 4 shows the confusion matrix of the fully fine-tuned MatSciBERT model. It can be seen that the model misses out on predicting the class “Mechanism” the most, 81 entities of type “Mechanism” were predicted as “O” (Outside)-tokens, i.e. background, by the model. Another interesting confusion shown by the model is predicting entities of type “Defect” and “Microstructure” as “Damage”. The confusion between “MechanicalTestParameter” and “PhysicalQuantity” is another notable confusion present in the model. The origin of these confusions will be analyzed in the discussion section.

## Discussion

### In-context learning of foundation models.

One high-level observation from Fig. 2 is that our two-stage prompting of foundation LLMs does not perform as well as the fine-tuned task-specific NER models on both in-distribution and out-of-distribution datasets. This could be attributed to several factors, like ICL struggling to grasp domain-specific terminology and patterns in materials science. Fine-tuning permits this by explicitly adapting the model parameters based on the provided training examples. However, it is not clear whether ICL can achieve the same since the model weights are not explicitly altered. Dai et al.^33^ describe ICL as a meta-optimizer that could implicitly modify the attention weights of a transformer-based LLM based on the provided few-shot demonstrations. However, whether such modifications can bridge larger domain gaps between pre-training and downstream task domains is not well studied. Xie et al.^34^ attribute the effectiveness of ICL to the presence of long-range coherence in the pre-training documents that forces LLMs to learn so-called latent concepts shared across sentences during pre-training. They explain that ICL works if the demonstrations in the prompt could be used by the LLM to identify the required latent concept for solving the task. In our NER setting, the required latent concept corresponds to the distribution of words representing a specific entity type as well as the relation of those words to other co-occurring words. Specifically, ICL works if the probability distribution of the LLM’s output is sharpened on the latent concept through common structures in demonstrations provided in the ICL prompt. However, this theory suggests that the latent concept is learned during pre-training, which might not be the case for domain-specific entities. With respect to the presented NER task, this implies that ICL could correctly identify entities that are more general but would fail on materials science-specific entities. Indeed, in Fig. 3, such a behavior can be observed. The pre-training domain being a decisive factor is also supported by the fact that, on a generic domain NER task, the effectiveness of ICL with a GPT-3.5 Turbo LLM was demonstrated by Wang et al. where the ICL performance was approaching that of state-of-the-art NER models^25^.

Moreover, the latent knowledge and ICL capabilities acquired by LLMs during pre-training could distinguish the performance gap between GPT-4 and GPT-3.5 Turbo, as evident in Fig. 2. Though exact information is not disclosed, GPT-4 is presumed to be pre-trained with comparatively more sophisticated training routines on a significantly larger corpus compared to GPT-3.5 Turbo. The larger corpus could have potentially helped GPT-4 acquire better domain-specific semantic priors. Additionally, the larger model size could enable GPT-4 to learn better from demonstrations in an ICL setting, which is consistent with inferences of Wei et al.^35^. The capability of GPT-4 to process and execute instructions in the prompt was also observed incidentally in the study. As depicted in Fig. 6, we explicitly specified in the task description not to generate output if there are no named entities of the specified type in the target sentence. It was observed that GPT-4 adhered to the instructions contrary to GPT-3.5 Turbo, which frequently repeated the target sentence without any annotations for cases where no named entities were detected.

Another reason for the mediocre performance of the two-stage ICL approach is that only ten few-shot examples per category were provided—though arguably the most relevant. In contrast, Fig. 3 states the substantially larger number of training examples provided per category for fine-tuning. Adding more few-shot demonstrations in the prompt has been shown to improve the performance of ICL on the NER task in a general domain^25^. Intuitively, this should be even more applicable in domain-specific settings. However, adding more context would also result in an increased number of input tokens and thus more expensive model inferences. The discrepancy in performance may also stem from the inherent limitations in detecting and classifying nuanced entity spans. This is addressed in recent developments in two-stage prototypical networks by Guo et al.^36^ by introducing boundary-aware contrastive learning to enhance span detection. Additionally, they employ domain-adaptive prototype alignment to ensure more consistent classification across source and target domains.

**Fig. 5:**
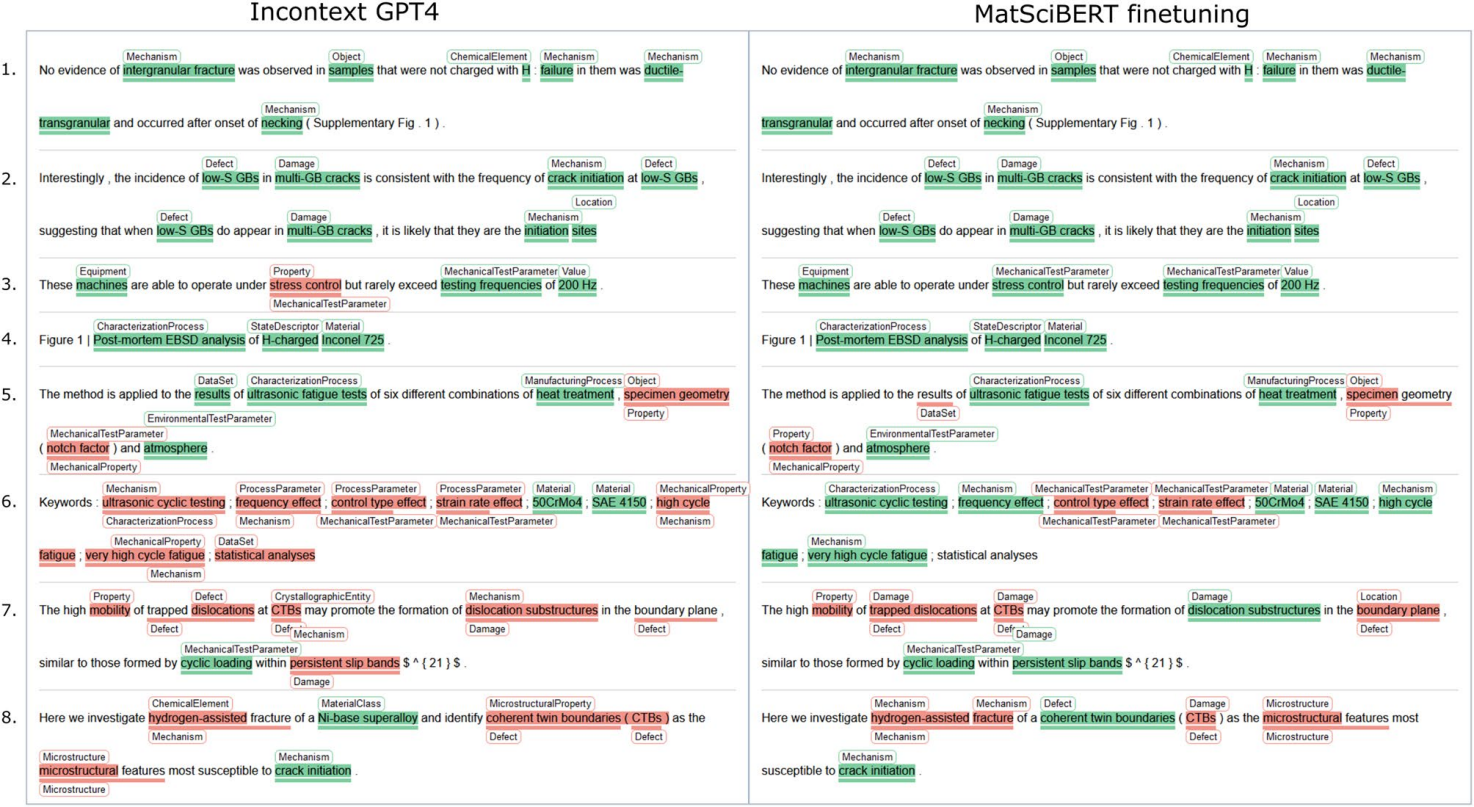
Predictions for multiple sentences where the in-context GPT-4 model and the best seed of the fine-tuned MatSciBERT model are compared to the annotation. The box below and above the marked spans indicate the annotated and predicted class, respectively. The visualization is created using ELEVANT^37^. The detailed evaluation results for all models can be reviewed on this ELEVANT page.

Notably, the relative performance difference between the validation (in-distribution) and test (out-of-distribution) evaluations is more pronounced for our ICL approach. This indicates that foundation models, even though often presumed to generalize well, heavily depend on relevant demonstrations in such domain-specific settings assuming no model optimization toward the target domain is performed. The performance difference between both domains could originate from the domain gap, i.e. distinct input and/or label distributions. While the validation dataset has neither domain shift nor distinct author style with respect to the training dataset, both differences exist for the test dataset. In the current state, it is difficult to assess the quantitative contributions to performance through the (i) limited corpus, (ii) the quality of the retrieved demonstrations, (iii) and generation, i.e., transfer of the relevant information from the demonstrations to the target sentence by the LLM. Intuitively, retrieval of demonstrations from the training dataset should be easier for the validation case due to matching domain and style. As a quantitative metric for retrieval quality, the Euclidean distance between embedding vectors representing the target sentence and retrieved demonstrations can be used. In our case, the average Euclidean distances amounted to 0.347 and 0.333 for target sentences from test and validation, respectively. This marginal difference might be owed to the usage of an all-purpose embedding model which squashes content from materials mechanics into a smaller vector subspace without taking the distinct subdomain between test and validation into account sufficiently. Similarly, it is to be expected that the LLM can infer entities easier if the provided demonstrations originate from the same corpus. Increasing the base corpus in variance and an improved retrieval-strategy alongside domain-specific embedding models which retrieve semantically similar instances and are style-agnostic can facilitate a performance improvement.

### Limitations and future research

A couple of factors related to the design of the ICL prompt workflow could be detrimental to its performance. Firstly, in the detection prompt, each LLM call only asks it to predict entities belonging to a single entity type. Thus, at detection time, the existence of alternative entity types is not indicated by the prompt and consequentially this information is not provided to the LLM. Aside from this, the few-shot demonstrations for the searched entity type are fetched from a partition in the vector store which contains only sentences in which the target entity type occurs. Thus, no negative samples are provided within the prompt. The lack of alternative class mentions and negative demonstrations could incentivize the LLM to predict entity categories that are otherwise less likely to be predicted. In contrast, the fine-tuning approach learns from examples belonging to all categories which could culminate in rather well-defined decision boundaries. Moreover, the rule-based approach used for flattening of NER predictions cannot discern which of the individual entity type predictions are more likely since the probability values were not provided by OpenAI’s chat completion endpoint at the time of inference. Thus, the rule-based step to resolve overlapped predictions described in the methodology section could end up selecting an incorrect entity type candidate. Rather than flattening the output, prospectively, ICL can facilitate a nested NER approach to do justice to word groups embodying multiple meanings.

Further, special cases were observed in the token-level predictions where the correct entity type was predicted but the predicted span differed from the ground truth span. Such errors constitute a larger fraction of total predictions (12.13%) in ICL of GPT-4 compared to the best fine-tuned task-specific model MatSciBERT (10.64%). An example is illustrated in Fig. 5 example #7, where the in-context GPT-4 predicts the span of the ground truth entity ‘trapped dislocation’ wrong but predicts its type correctly. Similarly, in sentence #8, GPT-4 gets both type and span wrong for the ground truth entities ‘coherent twin boundaries’ and its abbreviation ‘CTB’. On the other hand, the fully fine-tuned MatSciBERT model could predict the span correctly ignoring the parenthesis of the abbreviation. These examples are instances showing specific annotation guidelines in action, which are not guaranteed to be present in the few-shot demonstrations and hence ICL might not adhere to these guidelines. Furthermore, there is a wide variety of acronyms and chemical symbols that may be used without its expansion in the example sentences, for instance ‘CTB’ in sentence #7. In such cases, approaches which provide further context could be envisioned, e.g., using the vector store for retrieving the full forms of acronyms and agentic approaches, where domain-specific class definitions are automatically retrieved from APIs and supplemented in the prompt. Supplementing such information benefits from linking entity types to an ontology, Such so-called knowledge augmentation has lead to significant performance improvements in the biomedical domain^24,38,39^.

Another observation from Fig. 5 examples #4 and #6 is that materials are detected successfully by both model variants. In example #6, the material names are contained in a keyword list which indicates that contextual information is not mandatory to identify materials but that some materials and standards such as SAE or EN as well as trade names are memorized or local correlations are learned (concatenated chemical elements $\rightarrow$ material). Unlike the unified nomenclatures in chemistry, materials science lacks universal notations^40^, leading to diverse representations for the same material. For instance, the trade name ‘Inconel 725’, associated with a nickel-chromium-molybdenum-niobium alloy, might be readily recognized, however, linking with materials numbers (2.4857) and a breakdown of chemical compositions (Ni(balance), Cr(19–22.5%), Mo(7–9.5%), Nb(2.75–4.0%)) can present significant hurdles. This difficulty stems from the inherent ambiguity and lack of context within these notations. The abbreviated forms often only contain information about major alloying elements, neglecting crucial information like the presence of other micro-alloying constituents. In this work, a rather small quantity of publications—and by extension alloy classes—was considered. Thus, the generalization to arbitrary alloy systems and non-metallic materials needs to be explored in the future. Assuming the model can reliably identify materials through characteristic patterns within materials nomenclature standards, an approach could be envisioned where the materials definitions are automatically validated against an additional database as well as harmonized and enriched.

In this work, the objective is to thoroughly annotate full-texts to retrieve specifics about investigated materials and underlying processes. However, a fundamental characteristic of scientific research is building upon past findings, leading to a complex web of interconnected knowledge. Typically, previous work is cited and described in an abridged form rather than explicitly detailing methodologies, material compositions, or experimental setups. Such fragmentation poses a significant challenge for LLMs attempting to understand the intricacies of material science research. A viable solution to this is constructing hierarchically-structured knowledge graphs which do not only capture the document structure but also the citation network, similar to efforts which have been performed in the graph RAG field ^41^.

### Specialized NER models

The three BERT-based models, used for fine-tuning in this work, have the same architecture and mainly differ in the data used to pre-train the models. BERT is pre-trained on generic domain data from Wikipedia and BookCorpus^42^ datasets. SciBERT uses data from scientific domains by pre-training on a subset of more than a million full-text documents retrieved through SemanticScholar^43^, including 18% of computer science papers and 82% of medical papers. MatSciBERT uses datasets closer to the fatigue domain, pre-training on a materials science corpus containing 40% words from papers on inorganic glasses and ceramics, and 20% each from metallic glasses, alloys, and cement. As can be seen in Fig. 2, SciBERT and MatSciBERT both significantly outperformed BERT by 2–4% F1-score. Since the models differ mainly by pre-training datasets, the performance increase can be attributed to domain-specific pre-training. Domain specific pre-training has been shown to be helpful for various downstream tasks in domains including biomedicine (BioBERT^44^), finance (FinBERT^45^), law (CaseHold^46^), as well as materials science (MatBERT^47^ and MatSciBERT). This is especially beneficial if the dataset used for the downstream task is smaller^47^. However, it is to be noted that the pre-training data closest to the materials fatigue domain, i.e., the MatSciBERT corpus, is not specific for this subdomain which makes the model struggle to identify fatigue-domain specific terminologies. This could also be the reason behind the modest performance improvements for MatSciBERT compared to SciBERT. The inclusion of scientific writing styles in the datasets used in MatSciBERT and SciBERT might be primarily causing the performance improvement compared to BERT which used a diverse pre-training dataset with varying writing styles.

Some common confusions of the best conventionally fine-tuned MatSciBERT illustrated in Fig. 4 also motivate the need for even more domain-specific pre-training. For instance, the wrong prediction of “Defect” and “Microstructure” as “Damage” highlights that the model cannot learn the fine-grained differences between these classes. This could also be complicated by the presence of ground truth entities of these types that contain common words. For example, ‘multi-GB cracks’ (Fig. 5 2.) and ‘dislocation substructures’ (Fig. 5 7.) are of type “Damage” while ‘low-S GBs’ (Fig. 5 2.) and ‘trapped dislocations’ (Fig. 5 7.) are “Defect” entities. Our reasoning on the distinction between these semantically close entity types is provided in the accompanying data set publication^30^. The model often confuses “PhysicalQuantity” with “MechanicalTestParameter,” likely due to the presence of overlapping terms and their semantic similarity. Words like ‘stress’, ‘strain’, and ‘temperature’ typically represent physical quantities or state variables. In contrast, terms such as ‘strain rate’, ‘stress amplitude’, and ‘testing temperature’ usually describe mechanical test parameters or process metadata. This kind of overlap is common in materials science texts and highlights the need for nested named entity recognition (NER) approaches to better handle such nuances. The model also struggles with the “Mechanism” entity type, as shown in Fig. 3. This can be attributed to the broad definition of the “Mechanism” class. During annotation, a wide range of semantically diverse entities were grouped under this category, which likely contributed to missed predictions and lower overall performance. This motivates the refinement of the sub-hierarchy of the “Mechanism” class in the materials mechanics ontology^31^ and replacing “Mechanism” entity type with more fine-granular entity types.

From Fig. 2, we observe a drop in the performance for LoRA fine-tuning of BERT, SciBERT, and MatSciBERT compared to their full fine-tuning. Additionally, the LoRA fine-tuning of BERT and SciBERT falls short of their full fine-tuning counterparts by a larger margin (as opposed to MatSciBERT). This observation is consistent with the inferences from Biderman et al.^48^ who showed that when adapting LLMs to domains far from their pre-trained domain, LoRA tends to underperform compared to full fine-tuning. LoRA is proposed based on the hypothesis that the perturbations of model weights required to fine-tune a pre-trained model to a target domain or task could be approximated by low rank matrices. However, this hypothesis will not hold if a tougher downstream task necessitates higher-order alterations to the model weights that cannot be approximated by the typical LoRA configurations. Further, it is not possible to set distinct ranks to different model weights to tackle distinct levels of adaptation required. Techniques such as AdaLoRA^49^ and SoRA^50^ address this limitation by adaptively finding optimal ranks for weight matrices in different layers. The limitations of LoRA also encourage the use of PEFT methods that outperform LoRA including DoRA^51^ which is an architectural improvement to LoRA, and hybrid PEFT methods^52,53^ that jointly utilize distinct PEFT methods for different layers of the models.

Additionally, the generalization of the task-specific models, measured by the relative decrease in performance from in-distribution to out-of-distribution examples, was shown to be higher for LoRA compared to full fine-tuning. This supports the results of Biderman et al.^48^ that LoRA models tend to forget less and hence are less likely to overfit the fine-tuned domain. This also highlights the known problem of catastrophic forgetting in full fine-tuning^54^. We also observed that the LoRA fine-tuning outperformed conventional fine-tuning for the DeBERTa model. The performance achieved by LoRA fine-tuning of DeBERTa, despite a pre-training which was not focused on the materials domain, is remarkable. In the conventional fine-tuning of DeBERTa, only the classification head of the model was fine-tuned, which cannot learn any new feature representations but only adapt the features to the provided task. A full fine-tuning of DeBERTa could potentially exceed MatSciBERT performance but is computationally intensive and hence not recommended in time or memory-constrained scenarios. The number of trainable parameters in our LoRA DeBERTa configuration is a mere 0.12% of the total trainable parameters in the original DeBERTa model. Furthermore, as previously discussed, the full fine-tuning of DeBERTa may be susceptible to overfitting more to the training domain. These factors encourage pursuing PEFT methods for larger specialized NER models like DeBERTa.

## Conclusions

Designing tailored materials for specific applications is limited by the vast search space of materials and process parameters. The data available in existing literature could alleviate this pain point but the data is often incomplete and is not presented in a reusable manner. Thus, connecting entities extracted from literature to materials-specific ontologies is one step toward systems that could convert literature data to semantic and structured representations. The study compared two alternate methodologies that could tackle this named entity recognition (NER) task in materials science, namely creating task-specific language models and repurposing foundation large language models (LLMs) using in-context learning (ICL). The latter approach is appealing in principle as it can circumvent major efforts in preparing large-scale NER-annotated materials datasets. Beyond NER, additional tasks like coreference resolution and relation extraction need to be handled to semantically structure literature data in materials science. The usage of foundation LLMs provides the convenience that a single model could be repurposed to handle all these sub-tasks rather than complex systems of task-specific language models, which are often prone to error propagation.

However, the conducted experiments show that ICL with LLMs displays moderate entity recognition performance overall. Considering the limited number of demonstrations provided, i.e., only ten per entity type, the results of GPT-4 are promising nonetheless. For the fine-grained distinction of domain-specific entity types, a strong influence of domain shift was observed, where few-shot demonstrations from a slightly shifted domain caused a major decline in performance. It was also observed that the performance degraded for rather domain-specific entity types. For instance, ICL exhibits proficiency in identifying general entity types such as EnvironmentalTestParameter, ChemicalElement, Value, etc. However, the performance of ICL is suboptimal on entity types that are more specific to the materials mechanics domain, like Damage and Defect, etc. It was also demonstrated that GPT-4 consistently outperformed GPT-3 for all entity types in both domains. This indicates the benefit of larger foundation models in conjunction with a larger and more comprehensive pre-training corpus. In conclusion, for tasks which do not need particularly fine-grained distinction of domain-specific entities, an ICL approach could be practical. This holds especially for the low data quantity scenario where only few annotated entities are available for each entity type. When annotated data is scarce, approaches can be envisioned that use the latest retrieval strategies based on vector searches to select a few representative sentences for the target corpus for subsequent annotation and few-shot prompting.

The alternate approach of using specialized fine-tuning techniques achieved comparatively better NER results—especially on domain-specific entity types. The performance improvement of SciBERT and MatSciBERT over BERT demonstrated that pre-training on a corpus closer to the target domain is advantageous. The comparison among different fine-tuning strategies showed that full fine-tuning is preferable over LoRA for adapting the models to a novel task and domain—particularly when large domain shifts between pre-training and test domains are concerned. However, the computational expense and the risk of overfitting in full fine-tuning motivate the exploration of better parameter efficient fine-tuning (PEFT) methods for low-data scenarios like those in materials science. Additionally, the LoRA-DeBERTa experiment suggests that larger task-specific language models are capable of generalizing well to the target domain in PEFT. In conclusion, full fine-tuning of smaller task-specific models has been demonstrated to provide optimal performance on datasets that are similar to the training distribution. In addition, PEFT of larger task-specific language models is identified as promising to produce models that generalize better to a target domain. Irrespective of the exact fine-tuning methodology, the study recommends the use of task-specific language models that are pre-trained on a corpus closer to the downstream domain. Similarly, it is conceivable that fine-tuning open-source foundation language models toward the materials domain would improve its entity recognition capabilities and reduce the necessity for few-shot demonstrations.

## Methods

### Data generation

Aside from the MaterioMiner data^30^, which was used as a test dataset, a corpus of peer-reviewed materials science publications was acquired from which training and validation data were derived. The objective was to collect a representative materials fatigue text dataset which captures many of the relevant entities within the domain. Another target was to generate a thoroughly annotated dataset, where many distinct sections were thoroughly annotated to enable training language models that permit information extraction beyond abstracts. A set of 47 full-text PDF documents of scientific articles were manually selected where the focus was on fatigue of steel and aluminum alloys and further filtered to contain information on S-N curves, i.e. a relationship between applied cyclic loads and sustained lifecycles.

The PDF documents were converted into machine-readable format using the IBM Deep Search platform^55^. The details of the PDF conversion pipeline are explained in related publications^56–59^ and summarized here. Deep Search uses multiple neural networks which are trained to extract bounding boxes or PDF cells generated by PDF printing commands. Next, the cells are classified into several labels including title, author, abstract, affiliation, subtitle, text, formula, table, image, caption, footnote, citation, keyword, etc. The information contained in the PDF cells and their predicted labels are jointly assembled in a machine-readable format. The final machine-readable document engenders multiple downstream use-cases like question-answering from documents^60^, universal information extraction^2^, or NER as in the present case.

These converted documents were utilized to filter and extract relevant information. Through API calls to the IBM Deep Search platform, paragraphs containing terms from the materials mechanics ontology were extracted. These extracted paragraphs were segmented into sentences. The annotation of this corpus was done identically to the procedure outlined in the MaterioMiner publication^30^. The plain text files of the extracted paragraphs along with the materials mechanics ontology were loaded into the INCEpTION annotation tool^61^, where relevant entities therein were annotated by the curator of the test set (MaterioMiner dataset) to ensure consistency. The same subset of 28 target classes (C = 28) of the ontology was used and annotated as foreground classes as previously applied in the MaterioMiner dataset. The output from INCEpTION was formatted in CoNLL 2002 NER format^62^, which is a widely employed format for NER tasks that uses a BIO tagging scheme^63^ for the annotations. The BIO tagging uses the ‘B-’, ‘I-’ prefixes, and ‘O’, to indicate the beginning, inside, and outside of an entity, respectively.

The dataset was subsequently split into a train and validation set comprising 80% and 20% of the corpus, respectively. Hence the training and validation datasets are in-distribution with respect to each other. In contrast, there is a slight domain shift to the Materiominer dataset (test set) which entailed scientific literature addressing the correlation between microstructure and structural materials properties. The emphasis on fatigue is slightly weaker in the test set.

The original sentence texts were encoded through vector embeddings using OpenAI’s text-embedding-ada-002 model which generates 1536-dimensional embedding vectors in order to populate a vector datastore. Embeddings are numeric vector representations that capture the content of the text semantically and for instance allow efficient retrieval of sentences resembling target sentences. A Milvus vector store (version 2.3) was implemented with a collection for each split subset (train, validation, and test). Each collection further featured partitions which are subdivisions created for each annotated entity type. Whenever a sentence contained an annotation for an entity type, the sentence was subjoined into the corresponding partition. As the indexing approach, InVerted File Flat (IVF_FLAT) with a number of clusters of 128 (nlist = 128) was used which is a quantization-based indexing approach^64^. The outcome is the original sentence text stored along with potential annotations, the embedding vector, and accompanying metadata in the vector store.

### In-context learning

We leverage the capabilities of LLMs to consider the relevant context within prompts when generating responses for our NER task. The overall process can be delineated as two distinct stages (i) detection and (ii) self-validation. Both stages rely on distinct prompt templates which were designed in accordance with OpenAI’s prompt engineering guidelines^65^. After the detection stage and after the self-validation stage, we employ a series of rule-based post-processing steps.

**Fig. 6:**
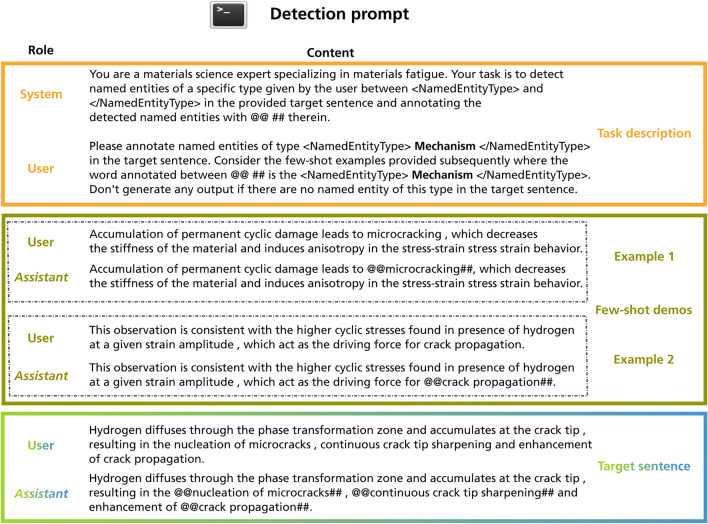
This figure shows the structure of the detection prompt. In the detection prompt, the LLM is requested to annotate the target entity using the provided special characters. This corresponds to binary token classification. The detection prompt is subdivided into three sections—the task description, the few-shot demonstration, and the target sentence. The colour-coding is associated with Fig. 1. Furthermore, the figure indicates the JSON format used by many OpenAI models where both role and content are defined. Depending on the intended behaviour of the LLM, messages can be conveyed through different roles. For brevity, not all few-shot demonstrations are shown. The last Assistant message represents the response of a model.

In contrast to the fine-tuned BERT models, which perform multi-class token classification, during ICL, we decompose the problem into multiple binary classification tasks where each foreground class is detected individually. This strategy is adopted from Wang et al.^25^ and their GPT-NER framework, which proposes to frame the NER task, which is a token-level classification task, as a text generation task in order to play to the strength of latest LLMs. This is achieved by bracketing named entities with special tokens @@ and ##. This approach presumably simplifies the task for the LLM, as it only needs to focus on replicating the text while marking the entity locations with the designated tokens. At the detection stage, the binary classification tasks are decoupled from each other. This means that the LLM is unaware of alternative classes when annotating for a specific class. The outcome of this stage is inherently non-flat NER.

**Fig. 7:**
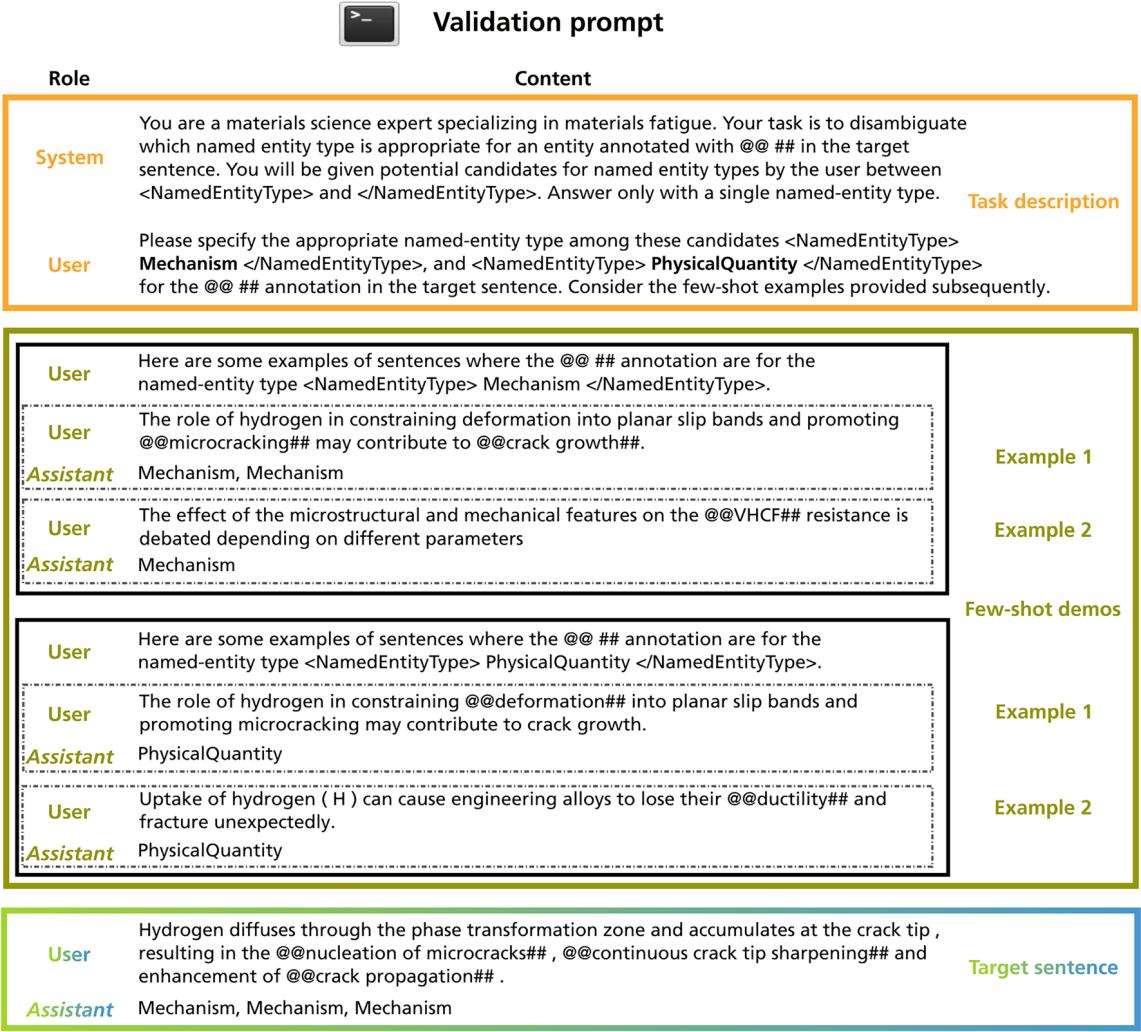
This figure shows the structure of the validation prompt. In the validation prompt, the LLM is requested to disambiguate entities which have been annotated more than once during the detection stage. The validation prompt is subdivided into three sections—the task description, the few-shot demonstration, and the target sentence. Furthermore, the figure indicates the JSON format used by many OpenAI models where both role and content are defined. Depending on the intended behaviour of the LLM, messages can be conveyed through different roles. For brevity, not all few-shot demonstrations are shown.

#### Detection of named-entity candidates

The prompt that we use at the detection stage is shown in Fig. 6. At a high-level, the prompt consists of three parts, namely, task-specification, few-shot demonstration, and a target sentence. We use the OpenAI’s chat completion API endpoint and adhere to the required format in the JSON bodies. The request body in their JSON format is partitioned into multiple roles, (i) system, (ii) user, and (iii) assistant which are each used to convey specific information to the LLM in a structured fashion^66^. The system role initiates the conversation by defining a persona and role which the LLM is supposed to fulfill. In our system message, we provide information about the domain of interest to the model to incentivize it to rely on the relevant portion of its latent “knowledge”. We use an XML tagging approach where classes of interest are referenced and demarcated in the prompt as XML elements since LLMs can understand such syntax to detect instance boundaries^65,67^. The user message within the task description section provides the target class to be detected and instructs the model to consider the subsequently provided few-shot examples. The most relevant few-shot examples from the train set are fetched based on their semantic similarity to the target sentence embedding vector from either the validation or test set. This is performed through an approximate nearest neighbor (ANN) search based on the Euclidean distance (L2-norm) metric which approximately identifies and retrieves the $k_1$-nearest neighbors, where $k_1$=10. The ANN search is restricted to the partition within the vector store that contains sentences with annotated instances of the target class. The few-shot demonstrations are crucial as they serve as a means to provide related domain-specific examples to the LLM which is otherwise unaware of our class definitions and annotation policies. The input and output of few-shot pairs are provided through messages alternating between user and assistant roles, respectively. This is in line with OpenAI recommendations^65^. Finally, we provide the target sentence from the validation or test set as a user message, inducing the LLM to generate an annotated version as output.

Decomposition into multiple binary classification tasks is motivated by the inherent input token limitations of the employed LLMs. Specifically, gpt-35-turbo-0301 and gpt-4-1106-preview were used which have a limit of 4096 and 128,000 input tokens, respectively, which affects the information that can be provided within a prompt. This renders it impractical to include comprehensive descriptions and demonstrations for all entity types within a single prompt. Furthermore, with exceedingly lengthy prompts and mixed entity type demonstrations, finding relevant information for generation might become increasingly difficult which is commonly denoted as needle in a haystack problem^68^. Consequently, for each input sentence, we construct the prompt *N* times, with each iteration corresponding to a distinct entity type.

#### Post-processing and self-validation

Following the detection phase, the identified candidate named entities within each sentence undergo post-processing to flatten the NER annotations, improve accuracy, and align with the annotation guidelines pre-defined by the involved dataset annotators. This process involves several key steps:

1. *Extracting entity spans* Start and end character positions (spans) are extracted for each detected entity within the original sentence.
2. *Identifying unique entities* If a span is uniquely annotated by the model, i.e. there are no overlaps between the binary classification iterations, the span is assigned the corresponding entity type.
3. *Resolving overlapped preditions* For non-uniquely annotated entities, we differentiate between three scenarios for further rule-based processing. The approach prioritizes the detection of longer spans, i.e. the largest entity mentioned is prioritized based on the Materiominer annotation guidelines^30^. The rule-based processing is described below:
  *Complete Overlap* If one entity completely encompasses another, i.e. represents a superset, the shorter prediction is discarded.
  *Partial Overlap* In cases of partial overlap, i.e. when both entities cover some distinct areas as well, the entity with the longest word span is retained.
  *Exact span match* When the same span is identified for two different entity types, these conflicting entities are presented to a separate validation prompt, which is designed to guide the LLM to select the most appropriate entity type.

The validation prompt is illustrated in Fig. 7 and has the same high-level structure as the detection prompt since it is partitioned into task description, few-shot demonstration, and target sentence. The task description outlines the specific objective of resolving entities that have received multiple annotations. Therefore, a list of candidate entity type labels is provided which were found at detection time. The LLM is tasked with leveraging the provided context and the $k_2$ few-shot demonstrations to determine the most appropriate entity type for conflicting annotations. The few-shot demonstration section provides $k_2=3$ exemplary instances from each entity type under consideration ($C_{val}$, with $C_{val} \subseteq C$). This serves as a reference for the LLM to obtain demonstrations of the desired output format and domain-specific information. Chat history from the detection phase is not propagated to the validation phase.

Once the LLM has resolved the conflicting annotations, we proceed to consolidate all detected entities for the target sentence into a final, consistent output that adheres to the BIO tagging scheme used for the ground truth.

### Conventional and parameter-efficient fine-tuning

The pre-trained language models were adapted to the NER task by concatentating the model with a classification head, which is a fully connected neural network layer with outputs corresponding to the B- and I- tags for each of the 28 entity types and the O-tag. Thus, the classification head contained 57 output neurons. The pre-trained weights of the language models were loaded from Hugging Face^14,69–71^, and the classification head was initialized with random weights. All the model weights were trained during full fine-tuning of all the BERT-based NER models. However, in the last-layer fine-tuning of the DeBERTa model, only the classification head was trained, keeping all other layer weights fixed.

LoRA^16^ was the parameter-efficient fine-tuning method adopted in this work. Here, the perturbation to weight matrices of the base model during fine-tuning is approximated using matrices of lower rank (called update matrices) using low-rank decomposition. During LoRA fine-tuning, only the update matrices are altered keeping the pre-trained weight matrices fixed, and subsequently the matrices are merged. In the LoRA training of all four pre-trained models, the query and value projection matrices of all attention layers were adapted using LoRA, the same as in the default setting of the original implementation. Among other weights, only the classification head weights are updated during the training.

All trainings used a cross-entropy loss with a AdamW^72^ optimizer with ß$_1$ = 0.9, ß$_2$ = 0.999, and $\epsilon = 1.10^{-8}$. The LoRA fine-tunings required additional parameters like rank of the matrices, scaling factor alpha, and dropout probability for the LoRA layers. A fixed rank of 8 is chosen considering previous findings that higher ranks result in minor performance improvements and could result in increased memory requirements^48^. The alpha is kept to be the same value as rank as suggested in the original LoRA paper^16^. An initial set of experiments were performed to fix some hyper-parameters, selecting a batch size of 32 and cosine-with-restarts learning rate scheduler. A hyper-parameter optimization search was then performed to find the weight decay and initial learning rate for each of the conventional fine-tuned models. Therefore, the Ray Tune^73^ implementation of the Bayesian optimization and hyper band (BOHB)^74^ algorithm was used, which combines probabilistic sampling in the hyperparameter space with bandit-based early stopping. The hyperparameter search for the LoRA fine-tunings additionally tuned the dropout probability. Both variants of fine-tuning were performed on a Nvidia Tesla V100 GPU.

#### Evaluation of models

All model performances are assessed using F1-score, which is defined as seen in Eq. (1):

1 $$\begin{aligned} \text {F1-score}&= 2 \cdot \frac{\text {Precision} \cdot \text {Recall}}{\text {Precision} + \text {Recall}}. \end{aligned}$$

The F1-score for each of the entity type was calculated using the formula above, and a micro average of the F1-score is computed to evaluate the model performance. For each entity type, the F1-score was computed based on the criterion of exact span and type matches for the named entity mention using the Seqeval^75^ package. This means a true positive is only when both span and type for a given predicted entity matches with the target annotation.

## Data Availability

The test dataset used in the study is the MaterioMiner dataset^30^, which is publicly accessible under the 10.24406/fordatis/329.2. The publications used to create the training and validation data are not licensed to be redistributed. Hence, the training and validation datasets are not made publicly accessible.

## Code availability

The underlying code for task-specific NER models and in-context learning of LLMs are available at https://gitlab.cc-asp.fraunhofer.de/iwm-micro-mechanics-public/code/materials-ner.

## References

[1] Xu, D. et al. Large language models for generative information extraction: A survey. *Front. Comput. Sci.* **2312**, 17617 (2024).

[2] Mishra, L. et al. Statements: Universal information extraction from tables with large language models for esg kpis. Preprint at http://arxiv.org/abs/2406.19102 (2024).

[3] Sayeed, H. M., Mohanty, T. & Sparks, T. D. Annotating materials science text: A semi-automated approach for crafting outputs with gemini pro. *Integr. Mater. Manuf. Innov.* **13**, 445–452. 10.1007/s40192-024-00356-4 (2024).

[4] Venugopal, V. & Olivetti, E. Matkg: An autonomously generated knowledge graph in material science. *Sci. Data* **11**, 217 (2024). 38368452 10.1038/s41597-024-03039-z PMC10874416

[5] Venugopal, V. & Olivetti, E. Matkg-2: Unveiling precise material science ontology through autonomous committees of llms. In *AI for Accelerated Materials Design-NeurIPS 2023 Workshop*.

[6] Dagdelen, J. et al. Structured information extraction from scientific text with large language models. *Nat. Commun.* **15**, 1418 (2024). 38360817 10.1038/s41467-024-45563-x PMC10869356

[7] Guarino, N., Oberle, D. & Staab, S. What is an ontology? *Handbook on Ontologies* 1–17 (2009).

[8] Ciatto, G., Agiollo, A., Magnini, M. & Omicini, A. Large language models as oracles for instantiating ontologies with domain-specific knowledge. Preprint at http://arxiv.org/abs/2404.04108 (2024).

[9] Vaswani, A. et al. Attention is all you need. *Adv. Neural Inf. Process. Syst.* **30**, 1 (2017).

[10] Devlin, J., Chang, M.-W., Lee, K. & Toutanova, K. Bert: Pre-training of deep bidirectional transformers for language understanding. http://arxiv.org/abs/1810.04805 (2019).

[11] Liu, Y. et al. Roberta: A robustly optimized bert pretraining approach. Preprint at http://arxiv.org/abs/1907.11692 (2019).

[12] He, P., Liu, X., Gao, J. & Chen, W. Deberta: Decoding-enhanced bert with disentangled attention. http://arxiv.org/abs/2006.03654 (2021).

[13] Beltagy, I., Lo, K. & Cohan, A. Scibert: A pretrained language model for scientific text. http://arxiv.org/abs/1903.10676 (2019).

[14] Matscibert. https://huggingface.co/m3rg-iitd/matscibert (Accessed 25 October 2023).

[15] Ding, N. et al. Parameter-efficient fine-tuning of large-scale pre-trained language models. *Nat. Mach. Intell.* **5**, 220–235. 10.1038/s42256-023-00626-4 (2023).

[16] Hu, E. J. et al. Lora: Low-rank adaptation of large language models. http://arxiv.org/abs/2106.09685 (2011).

[17] Pu, G., Jain, A., Yin, J. & Kaplan, R. Empirical analysis of the strengths and weaknesses of peft techniques for llms. Preprint at http://arxiv.org/abs/2304.14999 (2023).

[18] Liu, C., Zhang, W., Zhao, Y., Luu, A. T. & Bing, L. Is translation all you need? A study on solving multilingual tasks with large language models. Preprint at http://arxiv.org/abs/2403.10258 (2024).

[19] Jeong, S., Baek, J., Cho, S., Hwang, S. J. & Park, J. C. Adaptive-rag: Learning to adapt retrieval-augmented large language models through question complexity. Preprint at http://arxiv.org/abs/2403.14403 (2024).

[20] Truhn, D., Reis-Filho, J. S. & Kather, J. N. Large language models should be used as scientific reasoning engines, not knowledge databases. *Nat. Med.* **29**, 2983–2984 (2023). 37853138 10.1038/s41591-023-02594-z

[21] Wang, Y. et al. Exploring the reasoning abilities of multimodal large language models (mllms): A comprehensive survey on emerging trends in multimodal reasoning. Preprint at http://arxiv.org/abs/2401.06805 (2024).

[22] Wang, L. et al. A survey on large language model based autonomous agents. *Front. Comput. Sci.* **18**, 186345 (2024).

[23] Lu, Y. et al. Unified structure generation for universal information extraction. Preprint at http://arxiv.org/abs/2203.12277 (2022).

[24] Munnangi, M. et al. On-the-fly definition augmentation of llms for biomedical ner. Preprint at http://arxiv.org/abs/2404.00152 (2024).

[25] Wang, S. et al. Gpt-ner: Named entity recognition via large language models. Preprint at http://arxiv.org/abs/2304.10428 (2023).

[26] Kasner, Z. & Dušek, O. Beyond traditional benchmarks: Analyzing behaviors of open LLMs on data-to-text generation. In *Proceedings of the 62nd Annual Meeting of the Association for Computational Linguistics (Volume 1: Long Papers)* (2024).

[27] Kasahara, T. & Kawahara, D. Exploring automatic evaluation methods based on a decoder-based llm for text generation. 10.48550/arxiv.2310.11026 (2023).

[28] Zheng, Z., Zhang, O., Borgs, C., Chayes, J. T. & Yaghi, O. M. Chatgpt chemistry assistant for text mining and the prediction of mof synthesis. *J. Am. Chem. Soc.* **145**, 18048–18062. 10.1021/jacs.3c05819 (2023). 37548379 10.1021/jacs.3c05819 PMC11073615

[29] Sequence generation with label augmentation for relation extraction. In *Proceedings of the AAAI Conference on Artificial Intelligence*, vol. 37, 13043–13050. 10.1609/aaai.v37i11.26532 (2023).

[30] Durmaz, A. R., Thomas, A., Mishra, L., Murthy, R. N. & Straub, T. An ontology-based text mining dataset for extraction of process-structure-property entities. *Sci. Data* **11**, 1112. 10.1038/s41597-024-03926-5 (2024). 39389990 10.1038/s41597-024-03926-5 PMC11467320

[31] Materials Mechanics Ontology. https://gitlab.cc-asp.fraunhofer.de/iwm-micro-mechanics-public/ontologies/materials-mechanics-ontology (Accessed 09 July 2024).

[32] Gupta, T. et al. MatSciBERT: A materials domain language model for text mining and information extraction. *NPJ Comput. Mater.* **8**, 102. 10.1038/s41524-022-00784-w (2022).

[33] Dai, D. et al. Why can gpt learn in-context? Language models implicitly perform gradient descent as meta-optimizers. Preprint at http://arxiv.org/abs/2212.10559 (2022).

[34] Xie, S. M., Raghunathan, A., Liang, P. & Ma, T. An explanation of in-context learning as implicit Bayesian inference. Preprint at http://arxiv.org/abs/2111.02080 (2021).

[35] Wei, J. et al. Larger language models do in-context learning differently. Preprint at http://arxiv.org/abs/2303.03846 (2023).

[36] Guo, Q. et al. BANER: Boundary-aware LLMs for few-shot named entity recognition. In *Proceedings of the 31st International Conference on Computational Linguistics* 10375–10389 (Association for Computational Linguistics, 2025).

[37] Bast, H., Hertel, M. & Prange, N. Elevant: A fully automatic fine-grained entity linking evaluation and analysis tool. Preprint at http://arxiv.org/abs/2208.07193 (2022).

[38] Ghosh, S., Tyagi, U., Kumar, S. & Manocha, D. Bioaug: Conditional generation based data augmentation for low-resource biomedical ner. In *Proceedings of the 46th International ACM SIGIR Conference on Research and Development in Information Retrieval* 1853–1858 (2023).

[39] Chen, P., Wang, J., Lin, H., Zhao, D. & Yang, Z. Few-shot biomedical named entity recognition via knowledge-guided instance generation and prompt contrastive learning. *Bioinformatics* **39**, 496 (2023). 10.1093/bioinformatics/btad496 PMC10444965 37549065

[40] Miret, S. & Krishnan, N. Are llms ready for real-world materials discovery? Preprint at http://arxiv.org/abs/2402.05200 (2024).

[41] Hu, Y. et al. Grag: Graph retrieval-augmented generation. Preprint at http://arxiv.org/abs/2405.16506 (2024).

[42] Zhu, Y. et al. Aligning books and movies: Towards story-like visual explanations by watching movies and reading books. Preprint at http://arxiv.org/abs/1506.06724 (2015).

[43] Ammar, W. et al. Construction of the literature graph in semantic scholar. In *Proceedings of the 2018 Conference of the North American Chapter of the Association for Computational Linguistics: Human Language Technologies, Volume 3 (Industry Papers)* 84–91. 10.18653/v1/N18-3011 (Association for Computational Linguistics, 2018).

[44] Lee, J. et al. Biobert: A pre-trained biomedical language representation model for biomedical text mining. *Bioinformatics* **36**, 1234–1240 (2020). 31501885 10.1093/bioinformatics/btz682 PMC7703786

[45] Liu, Z., Huang, D., Huang, K., Li, Z. & Zhao, J. Finbert: A pre-trained financial language representation model for financial text mining. In *Proceedings of The Twenty-Ninth International Conference on International Joint Conferences on Artificial Intelligence* 4513–4519 (2021).

[46] Zheng, L., Guha, N., Anderson, B. R., Henderson, P. & Ho, D. E. When does pretraining help? Assessing self-supervised learning for law and the casehold dataset of 53,000+ legal holdings. In *Proceedings of the Eighteenth International Conference on Artificial Intelligence and Law* 159–168 (2021).

[47] Trewartha, A. et al. Quantifying the advantage of domain-specific pre-training on named entity recognition tasks in materials science. *Patterns* **3**, 1 (2022). 10.1016/j.patter.2022.100488 PMC9024010 35465225

[48] Biderman, D. et al. Lora learns less and forgets less. Preprint at http://arxiv.org/abs/2405.09673 (2024).

[49] Zhang, Q. et al. Adaptive budget allocation for parameter-efficient fine-tuning. Preprint at http://arxiv.org/abs/2303.10512 (2023).

[50] Ding, N. et al. Sparse low-rank adaptation of pre-trained language models. Preprint at http://arxiv.org/abs/2311.11696 (2023).

[51] Liu, S.-Y. et al. Dora: Weight-decomposed low-rank adaptation. Preprint at http://arxiv.org/abs/2402.09353 (2024).

[52] Mao, Y. et al. Unipelt: A unified framework for parameter-efficient language model tuning. Preprint at http://arxiv.org/abs/2110.07577 (2021).

[53] Chen, J. et al. Parameter-efficient fine-tuning design spaces. Preprint at http://arxiv.org/abs/2301.01821 (2023).

[54] Kirkpatrick, J. et al. Overcoming catastrophic forgetting in neural networks. *Proc. Natl. Acad. Sci.* **114**, 3521–3526 (2017). 28292907 10.1073/pnas.1611835114 PMC5380101

[55] Pyzer-Knapp, E. O. et al. Accelerating materials discovery using artificial intelligence, high performance computing and robotics. *npj Comput. Mater.* **8**, 84 (2022).

[56] Pfitzmann, B., Auer, C., Dolfi, M., Nassar, A. S. & Staar, P. DocLayNet: A large human-annotated dataset for document-layout segmentation. In *Proceedings of the 28th ACM SIGKDD Conference on Knowledge Discovery and Data Mining*. 10.1145/3534678.3539043 (ACM, 2022).

[57] Auer, C., Dolfi, M., Carvalho, A., Ramis, C. B. & Staar, P. W. J. Delivering document conversion as a cloud service with high throughput and responsiveness. In *2022 IEEE 15th International Conference on Cloud Computing (CLOUD)*. 10.1109/cloud55607.2022.00060 (IEEE, 2022).

[58] Livathinos, N. et al. Robust pdf document conversion using recurrent neural networks. Preprint at http://arxiv.org/abs/2102.09395 (2021).

[59] Staar, P. W. J., Dolfi, M., Auer, C. & Bekas, C. Corpus conversion service. In *Proceedings of the 24th ACM SIGKDD International Conference on Knowledge Discovery & Data Mining*. 10.1145/3219819.3219834 (ACM, 2018).

[60] Mishra, L. et al. Esg accountability made easy: Docqa at your service. *Proc. AAAI Conf. Artif. Intell.* **38**, 23814–23816 (2024).

[61] Klie, J.-C., Bugert, M., Boullosa, B., de Castilho, R. E. & Gurevych, I. The inception platform: Machine-assisted and knowledge-oriented interactive annotation. In *Proceedings of the 27th International Conference on Computational Linguistics: System Demonstrations* 5–9 (Association for Computational Linguistics, 2018).

[62] Tjong Kim Sang, E. F. Introduction to the conll-2002 shared task: Language-independent named entity recognition. In *Proceedings of CoNLL-2002* 155–158 (2002).

[63] Ramshaw, L. A. & Marcus, M. P. Text chunking using transformation-based learning. In *Natural Language Processing Using Very Large Corpora* 157–176 (Springer, 1999).

[64] Moffat, A. & Zobel, J. Self-indexing inverted files for fast text retrieval. *ACM Trans. Inf. Syst.* **14**, 349–379 (1996).

[65] OpenAI. *Prompt Engineering*. https://platform.openai.com/docs/guides/prompt-engineering/strategy-write-clear-instructions (Accessed 22 June 2024).

[66] OpenAI. *Chart Completion* (n.d.).

[67] Sui, Y., Zhou, M., Zhou, M., Han, S. & Zhang, D. Table meets llm: Can large language models understand structured table data? A benchmark and empirical study. In *Proceedings of the 17th ACM International Conference on Web Search and Data Mining* 645–654 (2024).

[68] Machlab, D. & Battle, R. Llm in-context recall is prompt dependent. Preprint at http://arxiv.org/abs/2404.08865 (2024).

[69] Bert Base Model (Uncased). https://huggingface.co/bert-base-uncased (Accessed 25 October 2023).

[70] Scibert Scivocab Uncased. https://huggingface.co/allenai/scibert_scivocab_uncased. (Accessed 25 October 2023).

[71] Deberta-v2-xxlarge. https://huggingface.co/microsoft/deberta-v2-xxlarge (Accessed 25 October 2023).

[72] Loshchilov, I. & Hutter, F. Decoupled weight decay regularization. Preprint at http://arxiv.org/abs/1711.05101 (2019).

[73] *Running Tune Experiments with Bohb*. https://docs.ray.io/en/latest/tune/examples/bohb_example.html (Accessed 05 November 2023).

[74] Falkner, S., Klein, A. & Hutter, F. Bohb: Robust and efficient hyperparameter optimization at scale. In *International Conference on Machine Learning* 1437–1446 (PMLR, 2018).

[75] Nakayama, H. *seqeval: A Python Framework for Sequence Labeling Evaluation*. https://github.com/chakki-works/seqeval (2018).

